# IT- technology for vascular monitoring as an evidential base in prevention and prediction of a vascular pathology

**DOI:** 10.1186/1878-5085-5-S1-A64

**Published:** 2014-02-11

**Authors:** Ulyana B Lushchyk, Viktor V Novytskyy, Rostislav V Bubnov, Ivanna I Legka, Andriy O Kovpak

**Affiliations:** 1 Research Center Veritas, 31 Obolonska Str., of 9, Kyiv, 04071, Ukraine; 2 The Centre of ultrasound diagnostics and interventional sonography, Clinical hospital “Pheophania” of State Affairs Department, Kyiv, Ukraine; 3 Center for Innovative Medical Technologies Veritas IT Med, 4 Williams Str., Kyiv, 03191, Ukraine

## Scientific objectives

Today the situation with cardiovascular diseases becomes a threatening phenomenon not only in Ukraine but in many countries of the world [1,2]. In Ukraine the Veritas Research Center and the Clinic of Healthy Vessels have been elaborated some algorithms for multivector correction of angioarchitectonics and vascular channel’s functions on the basis of the vascular blood flow theory for 17 years.

## Technological approaches

The project aims at further development and realization of IT-technology for vascular monitoring:

1) express diagnostics and screening of cardiovascular dysfunction in population;

2) further diagnostics, prevention and treatment of the detected pathology;

3) creation of some personalized algorithms for monitoring of vascular channel reconstructions on principles of evidential medicine;

4) forming the systemic approach to the medical management of patients with the cardiovascular pathology.

## Results interpretation

The project realization has economic, social and medical effects:

1. **The economic** - decreasing of costs and treatment duration and increase of efficiency of the individually oriented treatment. For the first month of treatment the coefficient of treatment efficiency grows from 70% to 320% according to regress of the subjective feelings and objective parameters in comparison with standard treatment approaches.

2. **The medical** - stable treatment results, which can last for half-a-year-year without any symptomatic medicines.

3. **The social** - labor resources is provided without any disability. Prophylactic measures after vascular screening allows avoiding critical vascular conditions. The secondary personalized medical approaches under control of the vascular monitoring allow establishing the stable regime for the cardiovascular system without any reiteration of critical conditions.

## Outlook and expert recommendations

The vascular screening technology is an intellectual technology, which can be applied at medical establishments and does not need routine doctor’s work in the screening process. It is effective analytical information based on principles of evidential medicine and is necessary for administrative decisions in treatment [3]. It is used for the preventive personalized approach for solving patients’ problems.
